# MGMT downregulation by CRISPR/Cas13 RNA-guided RNA targeting enhances glioma cell sensitivity to TMZ chemotherapy

**DOI:** 10.1007/s11060-026-05500-y

**Published:** 2026-03-12

**Authors:** Terry J. Prins, Thomas J. Lai, Tie Li, Addison Fisher, Blaine S. C. Eldred, Ryan Mostafavi, Linda M. Liau, Robert A. Chong, Phioanh Leia Nghiemphu, Timothy F. Cloughesy, David A. Nathanson, Albert Lai

**Affiliations:** 1 Department of Neurology, University of California, Los Angeles, 10833 Le Conte Ave, BOX 957066, CHS 36-128, Los Angeles, CA 90095-7066 USA; 2 Department of Neurosurgery, University of California, Los Angeles, Los Angeles, CA USA; 3 Department of Medical and Molecular Pharmacology, University of California, Los Angeles, Los Angeles, CA USA

**Keywords:** Glioma, CRISPR, *MGMT*-methylation, Temozolomide, RNA-guided RNA targeting

## Abstract

**Background:**

Current standard of care for glioblastoma involves fractionated radiotherapy administered with Temozolomide (TMZ), a DNA-alkylating agent. Inhibition of the DNA repair enzyme, O⁶-methylguanine-DNA methyltransferase (MGMT), promotes sensitivity to TMZ, particularly in tumors that repress *MGMT* mRNA transcription through promoter methylation. Novel strategies to inhibit *MGMT* are a promising avenue to improve therapeutic outcomes to TMZ. We hypothesized that CRISPR-Cas13-mediated RNA regulatory silencing of *MGMT* mRNA enhances response of immortalized and primary patient-derived gliomaspheres to TMZ in vitro.

**Methods:**

We utilized the Cas13x and Cas13d variants to target *MGMT* mRNA in the MGMT-expressing LN18 glioma cell line and in two patient-derived gliomasphere lines (GS104, GS081). Cas13-guide RNA ribonucleoproteins were delivered via lipofection, and stable knockdown was achieved using a lentiviral all-in-one system. *MGMT* mRNA and protein downregulation were assessed by RT-PCR and Western blot, respectively. Cell viability and chemosensitivity to TMZ were evaluated using MTT assays.

**Results:**

Both Cas13x and Cas13d systems, directed by specific guide CRISPR RNAs, achieved rapid and potent knockdown of *MGMT* mRNA and protein in all tested cell lines. This downregulation of MGMT expression led to an increase in the cytotoxic effects of TMZ, sensitizing previously resistant glioma cells and patient-derived gliomaspheres to standard chemotherapy. The lentiviral Cas13d system established stable chemosensitization in gliomasphere models.

**Conclusion:**

CRISPR-Cas13-mediated targeting of *MGMT* mRNA is an effective strategy for overcoming TMZ resistance in in vitro glioblastoma models. This RNA regulatory editing approach offers a proof-of-principle for CRISPR mediated therapeutics in patients with *MGMT* unmethylated gliomas.

**Supplementary Information:**

The online version contains supplementary material available at 10.1007/s11060-026-05500-y.

## Introduction

Glioblastoma (GBM) is the most common and aggressive primary brain cancer in adults [1]. Current standard of care combines fractionated radiation with the DNA alkylating agent temozolomide (TMZ), which provides a modest improvement in overall survival [2, 3]. TMZ exerts its cytotoxic effect by methylating guanine residues in DNA; however, the DNA repair protein O⁶-methylguanine-DNA methyltransferase (MGMT) reverses this alkylation, resulting in chemoresistance [3, 4].

*MGMT* expression is epigenetically regulated by its gene promoter methylation status [5]. Clinical sensitivity to TMZ is confined to ~ 40% of patients with *MGMT* promoter methylation and resultant low MGMT levels [5]. Conversely, patients with an unmethylated *MGMT* promoter express high MGMT, leading to chemoresistance and worse outcomes [5]. Therefore, strategies to overcome MGMT-mediated chemoresistance are of therapeutic importance.

Previous efforts to inhibit *MGMT* have yielded mixed results. RNA interference (RNAi) studies were hindered by lack of specificity and complex in vivo delivery [6–8]. Furthermore, small molecule inhibitors designed to directly block MGMT protein activity failed to provide significant clinical benefit and have been associated with adverse side effects [9, 10]. More recently, CRISPR-based technologies have emerged as novel tools for modulating gene expression. Our group and others have demonstrated that dCas9-based epigenetic editing induces *MGMT* promoter methylation and enhanced TMZ sensitivity [11–14]. However, epigenetic approaches are limited by slow kinetics and in vivo delivery of the large dCas9-fusion protein [15].

To address these limitations, we turned to the Class 2, Type VI CRISPR-Cas13 system, which directly targets and degrades RNA transcripts more efficiently than epigenetic editing and with higher specificity than traditional RNAi [16]. In this study, we selected the Cas13x and Cas13d variants, which are distinguished by their high on-target efficiency and minimal non-specific collateral RNA degradation [11, 17]. We hypothesized that directing these Cas13 variants to degrade *MGMT* mRNA would effectively sensitize chemoresistant glioma cells to TMZ. Here, we demonstrate in vitro in both established glioma cell lines and patient-derived gliomaspheres that Cas13-mediated knockdown of *MGMT* significantly enhances the cytotoxic effects of TMZ chemotherapy, presenting a novel therapeutic strategy for glioblastoma.

## Materials and methods

For full reagent details and catalog numbers, please see Supplementary Table 4.

### Plasmids and construction

**Fig. 1:**
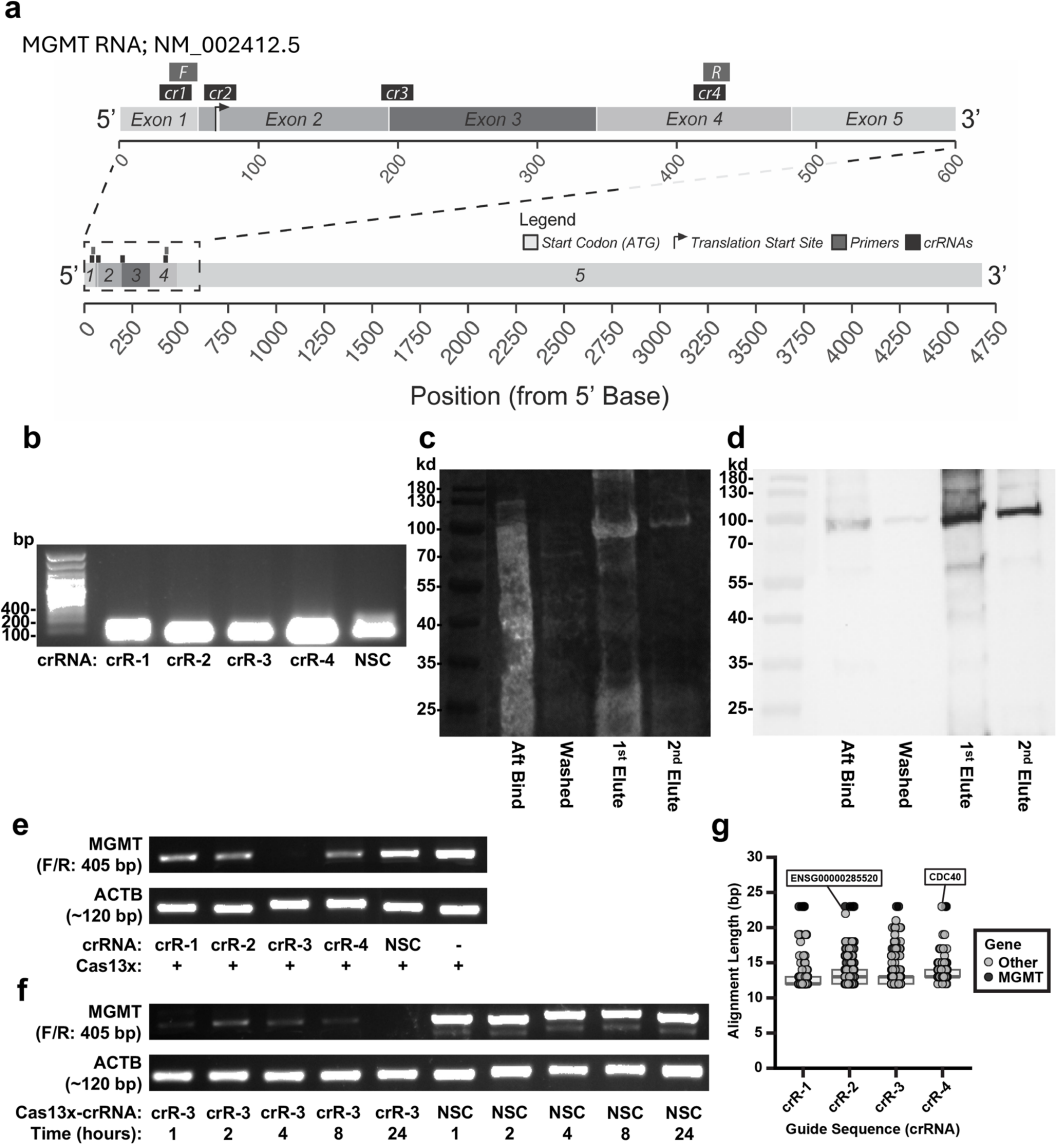
In vitro characterization and cleavage activity of the Cas13x/crRNA System Against *MGMT* mRNA. (**a**) Schematic of the *MGMT* mRNA transcript, indicating crRNA target sites and RT-PCR primer binding sites. (**b**) Agarose gel electrophoresis of the five in vitro transcribed crRNAs (NSC, *MGMT* crR-1, -2, -3, and − 4). (**c**) Ruby stain showing total purified Cas13x-His protein after electrophoresis and transfer to a nitrocellulose membrane. (**d**) Western blot of the same membrane from (**c**), probed with an anti-His antibody to confirm protein identity. (**e**) Cell-free cleavage assay. Total RNA from LN18 cells was incubated for 16-hours with pre-assembled Cas13x/crRNA ribonucleoprotein (RNP) complexes. Remaining *MGMT* mRNA was detected by RT-PCR (ACTB control). (**f**) Time-course of *MGMT* mRNA cell free cleavage by the Cas13x/crRNA-3 RNP. (**g**) Box-and-whisker plots for all crR-1, -2, -3, -4 (23 bp) NCBI BLAST+ results using the transcriptome as a reference, permitting mismatches and gapped (indels) alignments. A few potential “collateral” hits are highlighted

We used the pCMV-Cas13X.1 plasmid (Addgene) to express Cas13x and its corresponding guide CRISPR RNAs (crRNAs) in glioma cells. For cloning, we synthesized five pairs of DNA oligonucleotides (Supplementary Table 1) encoding a non-specific control (NSC) crRNA and four *MGMT*-targeting crRNAs (Fig. 1-a). The annealed templates were cloned into the *BbsI*-digested vector to generate five distinct Cas13x-crRNA expression plasmids, which were confirmed by DNA sequencing using BigDye Terminator v3.1 chemistry (Applied Biosystems).

For Cas13d and crRNA expression, we used the lentiviral plasmid pLentiRNACRISPR_006 (Addgene). We synthesized five pairs of DNA oligonucleotides (Supplementary Table 2) with the same crRNA sequences used for the Cas13x system. These were cloned into the *BsmBI*-digested vector to create five unique Cas13d-crRNA expression plasmids, validated by DNA sequencing.

### Cas13x protein expression in *E. coli* and purification

Recombinant Cas13x protein was produced by transforming the pET28-6His-SUMO-Nsp10 vector into *E. coli* BL21(DE3) and inducing expression with 100 µM IPTG. Cells were grown at 37 °C until 600 nm (OD₆₀₀) ~ 0.5, then incubated for 5 h post-induction. Cas13x-His protein was purified from the cell lysate under native conditions using a Ni Spin Column (NEB). Purity and protein identification was validated by SYPRO™ Ruby Protein Blot Stain (Invitrogen) and with Western blot with Rabbit Anti-His antibody (Proteintech).

### crRNA design, synthesis in vitro, and purification

The four *MGMT*-targeting crRNAs were identified and designed to prioritize target sites with high predicted RNA accessibility [18] and optimal GC content within the first 500 bases of *MGMT* mRNA (Fig. 1-a) [19]. We avoided regions with predicted high secondary structure complexity to ensure efficient binding of the Cas13-crRNA complex.

crRNA DNA templates, each including a 5’ T7 promoter sequence, were generated by PCR using five forward primers and one common reverse primer (Supplementary Table 3) and the Cas13x-crRNA-All-in-One vectors as template. The five crRNAs (NSC, *MGMT* crR-1, -2, -3, and − 4) were synthesized by in vitro transcription using the HiScribe™ T7 High Yield RNA Synthesis Kit (NEB). Products were purified with TRIzol™ reagent (Invitrogen) and validated by agarose gel electrophoresis.

For analysis of collateral activity, crRNAs were further evaluated for potential “off-target” sequence matches across the transcriptome (GENCODE v46) using Bowtie2 (v2.5.4, additional arguments: *–local –very-sensitive-local -L 10 -N 1 –rdg 3*,*1 –rfg 3*,*1 -k 100*) and the blastn algorithm from NCBI BLAST+ (v2.13.0, additional arguments: *-task blastn-short -word_size 4 -gapopen 2 -gapextend 1 -perc_identity 50 -evalue 700*).

### In vitro cleavage activity assay (cell free) of Cas13x/*MGMT*-crRNA ribonuclear protein (RNP)

The cell-free cleavage activity of the Cas13x RNP was measured following Konermann et al. [20]. Briefly, RNP complexes (1 µg Cas13x-His, 1 µg crRNA) were incubated with 2 µg of total RNA from LN18 cells in RNA cleavage buffer. Reactions were incubated at 37°C for specified time points (1, 2, 4, 8, or 24 hours). The reaction was terminated with TRIzol™ reagent, and the RNA was purified. Purified RNA was used to synthesize first-strand cDNA (SuperScript™ III Reverse Transcriptase, Invitrogen) and the remaining *MGMT* transcript was measured by PCR using the REDTaq^®^ ReadyMIX PCR Reaction Kit (Sigma-Aldrich) and *MGMT*-specific primers (Forward: 5’-TGCGCACCGTTTGCGACTTG-3’; Reverse: 5’-GGTTGCCTGCCAGGGCTGC-3’).

### Culture of LN18 glioma cells and patient derived glioma stem-like cells (gliomaspheres)

LN18 glioma cells were sourced from American Type Culture Collection (Manassas, Virginia) and primary patient-derived GS104 and GS081 gliomaspheres were supplied by Dr. Nathanson (Department of Molecular and Medical Pharmacology, UCLA). GS104 and GS081 were derived from newly diagnosed *IDH* wild-type WHO grade IV GBM with an unmethylated *MGMT* promoter and were selected due to their relatively fast growth in culture following transfection [21].

LN18 cells were maintained in DMEM (GIBCO) supplemented with 10% FBS and penicillin/streptomycin (37 °C, 5% CO₂) (GIBCO). Gliomaspheres were maintained in serum-free Neurobasal medium (GIBCO) supplemented with 1X Anti-Anti (GIBCO), 1X B27 (GIBCO), 5 µg/mL heparin (Sigma-Aldrich), 40 µg/mL EGF (Millipore), 40 µg/mL bFGF (Millipore), 1.25X N2 Supplement (GIBCO), and 200 mM L-Glutamine (GIBCO). All cell types express MGMT due to an unmethylated promoter and were cultured as previously described [11, 22].

### Cas13x transfection (LN18 cells)

Cas13x was transfected into LN18 cells as pre-assembled Cas13x-crRNA ribonucleoproteins (RNPs) using Lipofectamine™ CRISPRMAX™ Reagent (Invitrogen). For transient expression, the Cas13x-crRNA-All-in-One plasmid was transfected using X-tremeGENE™ HP DNA Transfection Reagent (Roche). Both were performed per the manufacturer’s protocols.

### Cas13d lentiviral transduction (LN18 and gliomaspheres)

To create stable cell lines, the Cas13d-crRNA-All-in-One lentiviral vector was first packaged into infectious lentiviral particles in HEK293T cells, as previously described [11, 22]. LN18 cells were then infected with the resulting virus. GS104 and GS081 gliomaspheres were infected using a spin-down method to enhance efficiency [22]. Three days post-infection, the cells underwent a five-day selection with puromycin (1.0 µg/ml for LN18; 0.7 µg/ml for gliomaspheres) to establish stable cell lines. Puromycin selected LN18 cells were grown out for one week before use in subsequent experiments while selected GS104 and GS081 cells required three months to establish stability.

### RT-PCR for *MGMT *mRNA

Total cellular RNA was isolated using the RNeasy^®^ Mini Kit (Qiagen). First-strand cDNA was synthesized from 0.5 µg of RNA using SuperScript™ III Reverse Transcriptase (Invitrogen). The resulting cDNA was used for PCR to quantify the relative abundance of the *MGMT* transcript.

### Western blot for MGMT protein and densitometry analysis

To assess MGMT protein levels, Western blot was performed as previously described [11]. The following primary antibodies were used: anti-HA, rabbit, (1:1000, Sigma); anti-MGMT, mouse (1:1000, ThermoFisher); GAPDH, mouse (1:2000, Proteintech). Densitometry analysis of relative MGMT protein expression was performed in *FIJI* (ImageJ) by normalizing MGMT band intensities to GAPDH (same gel) and using the *Analyze > Gels* function. All groups (NSC, crR-2, crR-3, and crR-4) were run together in each gel replicate, for a total of three replicates across two gliomasphere lines (GS104, GS081) and compared using a one-way permutation analysis of variance (ANOVA).

### Assay of cellular sensitivity to TMZ therapy

Cell viability following treatment was assessed using a standard MTT [3-(4,5-dimethyl-2-thiazolyl)-2,5-diphenyl-2 H-tetrazolium bromide] (Invitrogen) assay. Cells were seeded into 24-well plates (2,300 or 4,600 cells/well) and treated with varying concentrations of TMZ (50, 100, or 200 µM) or a DMSO vehicle control (4 days for LN18, 5 days for gliomaspheres). Following treatment, cells were incubated with MTT solution (0.5 mg/mL in regular culture media). The resulting formazan product was solubilized with DMSO, and absorbance was measured at 560 nm (background subtraction at 660 nm) to quantify cell viability.

### Statistical methods for MTT assays

Data were analyzed in Prism 9 via a student’s t-test or ANOVA with Tukey’s HSD post hoc test for multiple comparisons, where appropriate.

### Live-cell imaging

Representative phase-contrast images of gliomasphere cell lines were acquired using a Life Technologies EVOS XL Core Imaging System (Life Technologies, Cat# AME3302) and captured using a 10x LWD (long working distance) phase objective at room temperature. Quantification of cell radii from >10 live-cell images for each condition were performed in *FIJI* using the *Watershed* and *Analyze Particles* functions.

## Results

### Verification of *MGMT *mRNA cleaved by Cas13x/crRNAs in cell free condition

We first designed four unique *MGMT*-targeting crRNAs and a non-specific control (NSC) crRNA to target a 500-bp region near the 5’ end of the *MGMT* mRNA (Fig. 1-a). Their integrity was confirmed by agarose gel electrophoresis (Fig. 1-b).

The CRISPR-Cas13 family targets RNA [23]. We selected the Cas13x variant as our therapeutic effector due to its small molecular size, efficient on-target activity, and reduced off-target collateral activity [17]. We successfully expressed and purified the recombinant Cas13x protein from *E. coli* (Figs. 1-c, d).

To test the cleavage efficiency of our system, we assembled RNPs (purified Cas13x protein with synthesized crRNAs) and incubated them with total RNA from LN18 cells in a cell-free assay [24]. *MGMT* mRNA degradation was assessed by RT-PCR. All four *MGMT*-targeting RNPs efficiently cleaved the *MGMT* transcript compared to the NSC-RNP control (Fig. 1-e). The crRNA-3 RNP demonstrated the most potent cleavage activity. A time-course experiment revealed rapid cleavage by Cas13x/crRNA-3 RNP that occurred within the first hour (Fig. 1-f). To evaluate the selectivity of our system, we ran a highly permissive transcriptome-wide NCBI BLAST+ search for crR-1, -2, -3, and − 4, allowing for significant mismatches and gapped (indels) alignments (Fig. 1-g). No “off target” matching sequences were returned via a similar search with the Bowtie2 algorithm.

### Cas13x/crRNA-3 RNP transfection in LN18 cells knocks down MGMT expression and enhances TMZ sensitivity in vitro

**Fig. 2:**
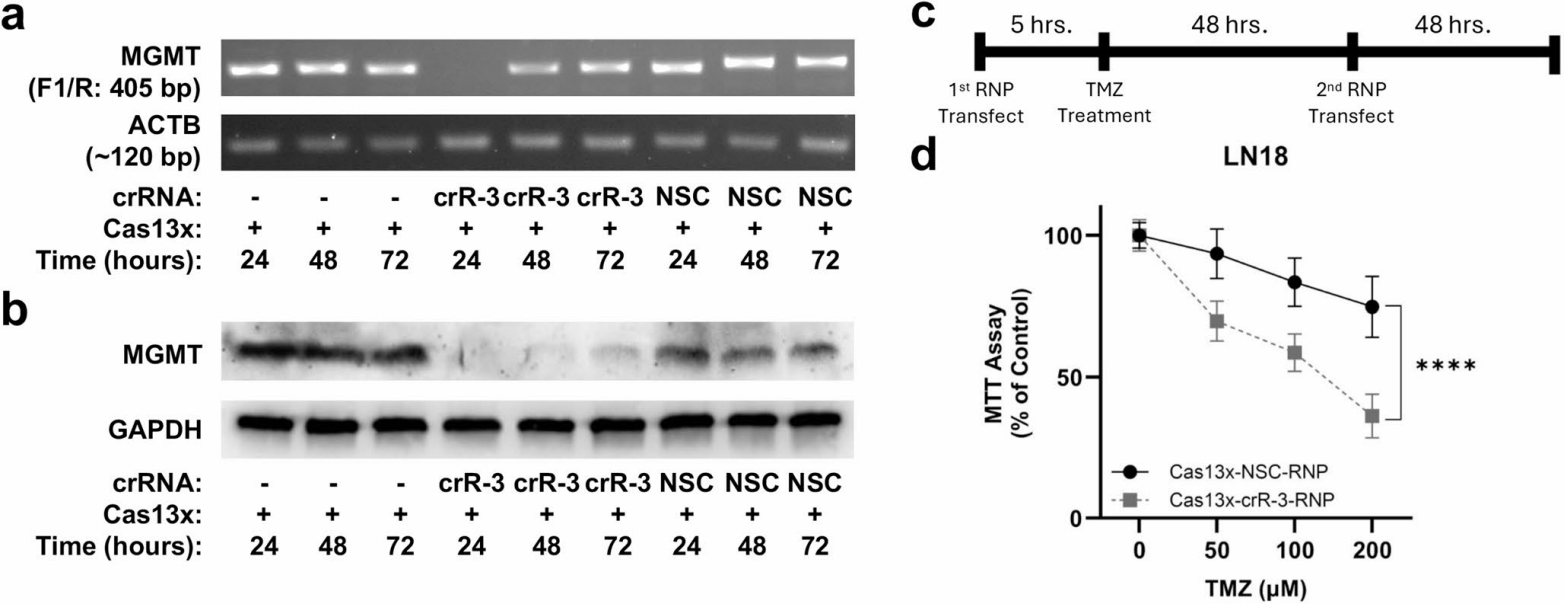
Cas13x/*MGMT*-crRNA RNP transfection reduces *MGMT* expression and sensitizes LN18 cells to TMZ. (**a**) Time-course of *MGMT* mRNA expression following RNP transfection (Cas13x protein alone, NSC RNP, or *MGMT *crR-3 RNP). *MGMT* mRNA levels were assessed by RT-PCR (ACTB control). (**b**) Time-course of MGMT protein levels following RNP transfection as in (a) assessed by Western blot (GAPDH control). (**c**) Dual RNP delivery strategy with TMZ treatment. (**d**) Cell viability of LN18 cells following dual RNP transfection and TMZ treatment (0, 50, 100, 200 µM) measured by MTT assay. Data represent the summary of three transfections (mean ± SEM). Statistical analysis: two-way ANOVA with Tukey’s post hoc test. [ANOVA, TMZ treatment: F(3,64) = 107.2, p ≤ 0.0001; NSC vs. crR-3: F(1,64) = 145.2, p ≤ 0.0001; TMZ treatment and crR-3/NSC interaction: F(3,64) = 19.65, p ≤ 0.0001. Asterisks indicate post hoc Tukey’s multiple comparisons test result for NSC vs. crR-3, summarizing results at all 3 TMZ conditions: 50, 100, and 200 µM]. ns, not significant, *,* p* ≤ 0.05; **, *p* ≤ 0.01; ***, *p* ≤ 0.001; and ****, *p* ≤ 0.0001

Given the potent guiding activity of crRNA-3, we proceeded with direct RNP transfection of LN18 cells. Knockdown of *MGMT* mRNA was nearly complete on day 1 post-transfection, with gradual recovery on days 2 and 3 (Fig. 2-a). A similar, slightly delayed reduction pattern was observed for MGMT protein, with recovery beginning after 72 h (Fig. 2-b).

Based on our previous work showing that a 4–5 day TMZ treatment is optimal for assessing viability changes in LN18 cells [11], we devised a double-delivery strategy to ensure sustained *MGMT* knockdown throughout the experiment (Fig. 2-c). The timing of the double-delivery strategy was optimized to prevent the recovery of down-regulated MGMT protein observed after 72 h of a single dose RNP dose (Fig. 2-b), thereby maximizing the targeting efficiency prior to assessing sensitivity to TMZ. LN18 cells treated with the Cas13x/crR-3 RNP showed a significant decrease in cell viability in the presence of TMZ compared to the non-specific control RNP (Fig. 2-d). These results demonstrate proof-of-concept for an RNA regulatory approach that sensitizes chemoresistant cells to TMZ using Cas13x.

### Lentiviral Cas13d system achieves stable MGMT knockdown in LN18 cells

**Fig. 3:**
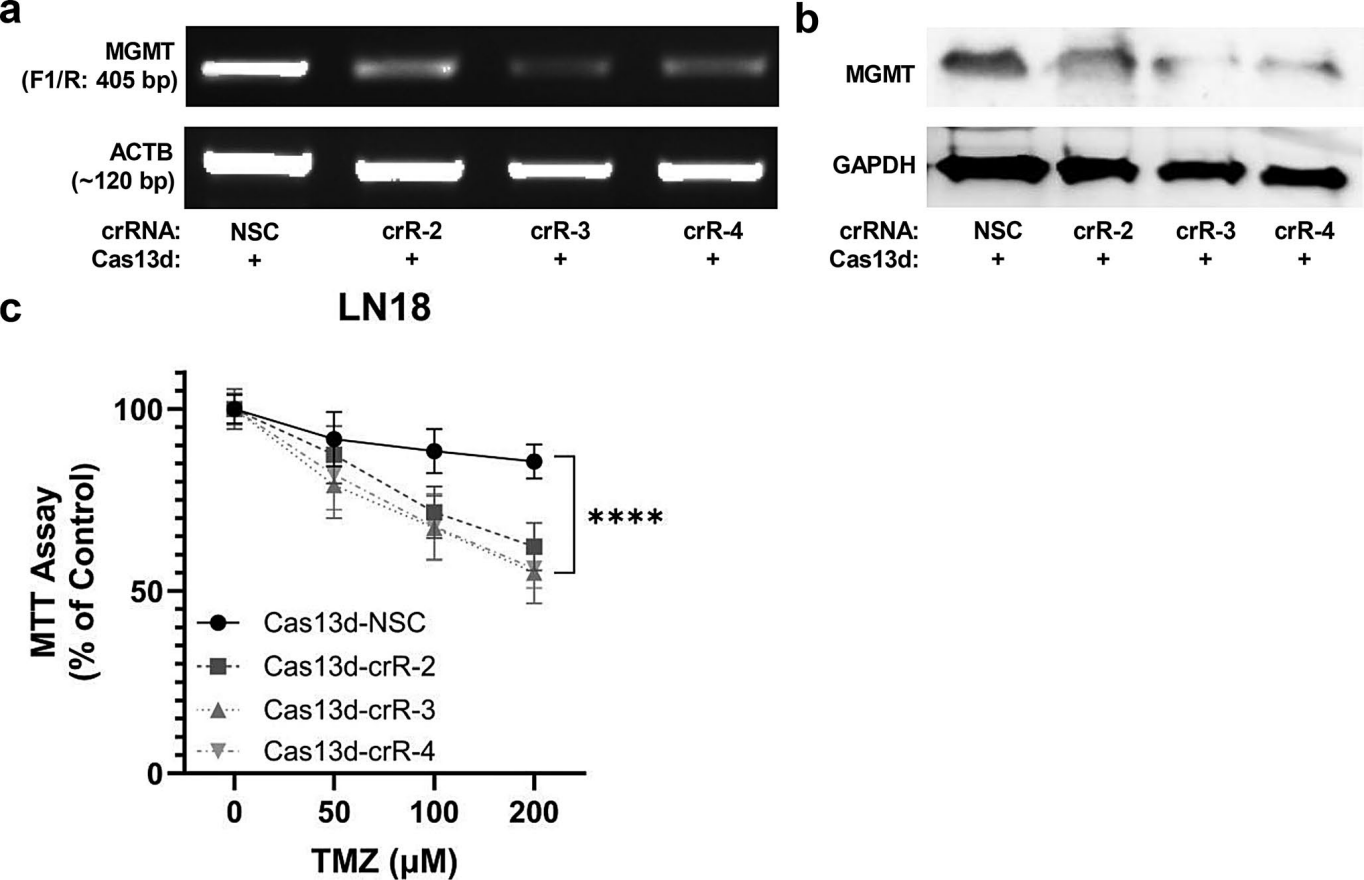
Lentiviral delivery of Cas13d/MGMT-crRNA stably reduces MGMT expression and sensitizes LN18 cells to TMZ. (**a**) RT-PCR of *MGMT* mRNA in transduced LN18 cells stably expressing Cas13d and crRNAs (ACTB control). (**b**) Western blot of MGMT protein in transduced LN18 cells (GAPDH control). (**c**) Cell viability (MTT assay) of stable cell lines after 96-hour treatment with increasing TMZ concentrations (0, 50, 100, or 200 µM). Data represent the summary of three transfections (mean ± SEM). Statistical analysis: two-way ANOVA with Tukey’s post hoc test. [ANOVA, TMZ treatment: F(3,188) = 236.0, p ≤ 0.0001; NSC vs. crR-2, -3, -4: F(3,188) = 53.74, p ≤ 0.0001; TMZ treatment and crR/NSC interaction: F(9,188) = 9.779, p ≤ 0.0001. Asterisks indicate post hoc Tukey’s multiple comparisons test result for NSC vs. each crRNA, summarizing results at all 3 TMZ conditions: 50, 100, and 200 µM]. ns, not significant, *,* p* ≤ 0.05; **, *p* ≤ 0.01; ***, *p* ≤ 0.001; and ****,* p* ≤ 0.0001

We next sought to establish a stable *MGMT* knockdown in LN18 cells using lentiviral vector delivery strategy. Given the lack of an available lentiviral vector for the Cas13x variant, we pivoted to the Cas13d system, which has a similarly high on-target efficiency and low collateral activity [17]. We constructed lentiviral vectors expressing Cas13d and crRNAs (NSC, *MGMT* crR-2, -3, and − 4). Following infection and selection, all three *MGMT*-specific crRNAs led to a marked downregulation of both *MGMT* mRNA and protein compared to the NSC control (Figs. 3-a, b). Consistent with Cas13x-crRNA RNP results, stably transfected Cas13d-crRNA elicited a significant increase in TMZ sensitivity in LN18 cells (Fig. 3-c).

### Cas13d lentiviral system sensitizes primary patient derived gliomaspheres to TMZ treatment

**Fig. 4:**
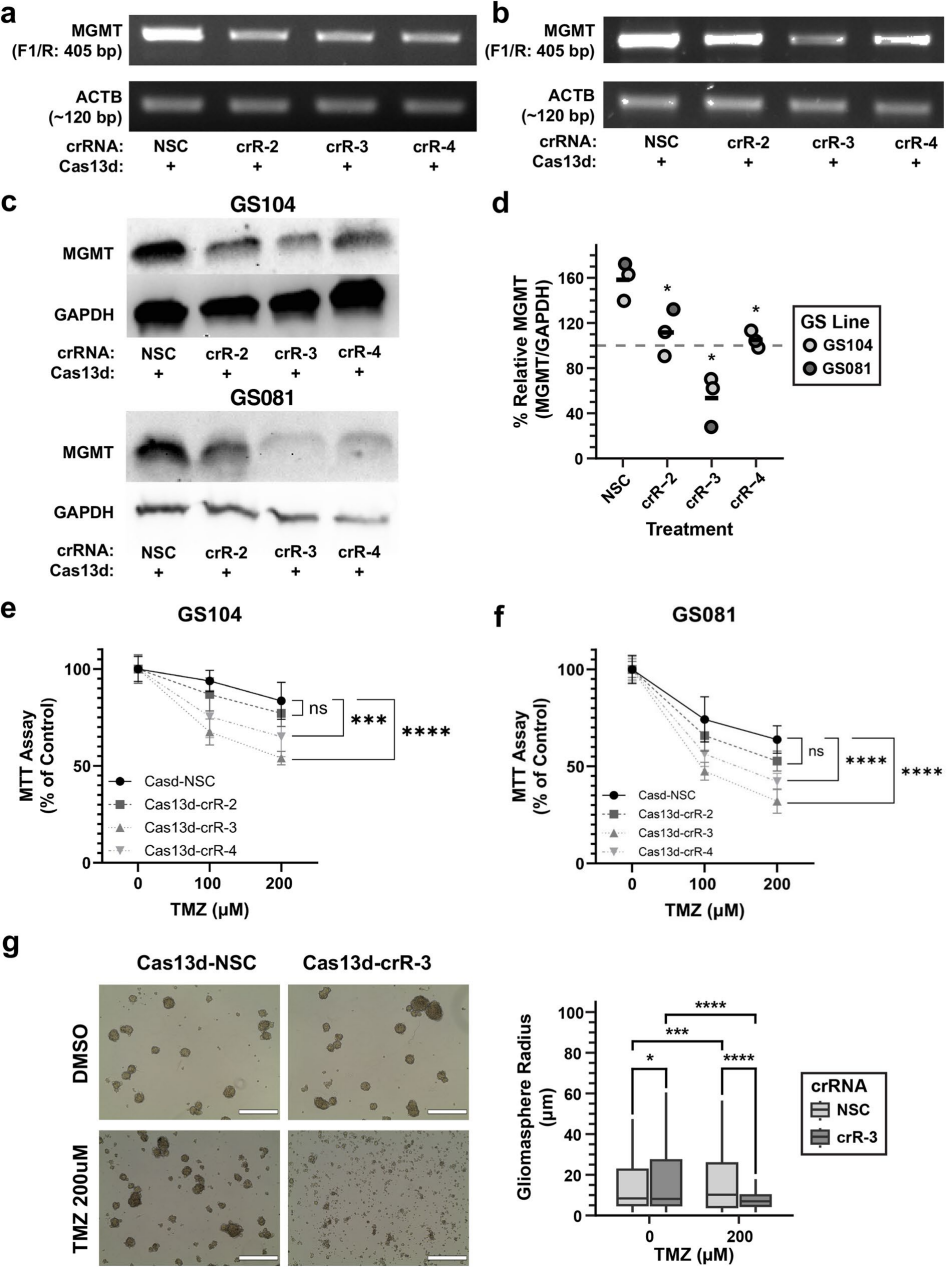
Cas13d-mediated knockdown of MGMT sensitizes patient-derived gliomaspheres to TMZ. (**a**, **b**) RT-PCR of *MGMT* mRNA in GS104 (**a**) and GS081 (**b**) gliomaspheres transduced with lentiviruses (*ACTB* control). (**c**) Western blot of MGMT protein in GS104 and GS081 gliomaspheres (GAPDH control). (**d**) Densitometry analysis of Western blot MGMT data from three independent experimental runs performed across two gliomasphere (GS104 and GS081) cell lines. All values are normalized to GAPDH expression; solid horizontal lines represent group means. Statistical analysis: one-way permutation ANOVA [ANOVA, RNA: F(3,8) = 17.31, p = 0.0008; asterisks indicate post hoc t-test result between crRNA and NSC with Benjamini-Hochberg multiple comparisons correction]. (**e**) Cell viability (MTT assay) of GS104 gliomaspheres after 96-hour treatment with increasing TMZ concentrations (0, 100, or 200 µM). Data represent the summary of three experiments from a single stable cell line (mean ± SEM). Statistical analysis: two-way ANOVA with Tukey’s post hoc test. [ANOVA, TMZ treatment: F(2,96) = 138.4, p ≤ 0.0001; NSC vs. crR-2, -3, -4: F(3,96) = 30.52, p ≤ 0.0001; TMZ treatment and crRNA/NSC interaction: F(6,96) = 7.743, p ≤ 0.0001; asterisks indicate post hoc Tukey’s multiple comparisons test result for NSC vs. each crRNA, summarizing results at both TMZ conditions: 100 and 200 µM]. (**f**) Cell viability (MTT assay) of GS081 gliomaspheres after 96-hour treatment with increasing TMZ concentrations (0, 100, or 200 µM). Data represent the summary of three experiments from a single stable cell line (mean ± SEM). Statistical analysis: two-way ANOVA with Tukey’s post hoc test. [ANOVA, TMZ treatment: F(2,60) = 394.5, p ≤ 0.0001; NSC vs. crR-2, -3, -4: F(3,60) = 28.26, p ≤ 0.0001; TMZ treatment and crR/NSC interaction: F(6,60) = 7.220, p ≤ 0.0001; asterisks indicate post hoc Tukey’s multiple comparisons test result for NSC vs. each crRNA, summarizing results at both TMZ conditions: 100 and 200 µM]. (**g**) Live-cell images of Cas13d-NSC-GS104 and Cas13d-crR-3-GS104 gliomaspheres after 14-day TMZ treatment (scale bars = 400 μm). Statistical analysis: two-way permutation ANOVA with post hoc Student’s t-test. [ANOVA, TMZ treatment: F(1,5724) = 227.81, p < 0.001; crR-3: F(1,5724) = 34.68, p < 0.001; TMZ treatment and crR-3/NSC interaction: F(1,5724) = 110.52, p < 0.001; asterisks indicate post hoc pairwise results with Holm method multiple comparisons correction]. ns, not significant, *, p ≤ 0.05; **, p ≤ 0.01; ***, p ≤ 0.001; and ****, p ≤ 0.0001

We applied the Cas13d-crRNA lentiviral system to the primary patient-derived gliomasphere models GS104 and GS081. Stable populations were generated expressing Cas13d with an NSC or one of the *MGMT*-targeting crRNAs (crR-2, -3, or -4). All three *MGMT*-targeting crRNA vectors reduced both *MGMT* mRNA (Figs. 4-a, b) and protein levels (Figs. 4-c, d) with crR-3 demonstrating the most potent suppressive effect on both lines (Fig. 4-d). Assessing translational relevance, treatment with TMZ (100 and 200 µM) showed a significant increase in TMZ-induced cytotoxicity in gliomaspheres expressing *MGMT* crRNAs compared to NSC controls (Figs. 4-e, f). crR-3 expressing spheres showed the greatest enhancement of TMZ sensitivity. Additionally, long-term 14-day treatment of Cas13d-crR-3 GS104 gliomaspheres with 200 µM TMZ resulted in a greater cytotoxic response, as visualized by live-cell imaging (Fig. 4-g).

## Discussion

In this study, we demonstrated that targeting *MGMT* mRNA with the CRISPR-Cas13 system effectively reverses TMZ chemoresistance in preclinical in vitro models of *MGMT*-unmethylated glioblastoma. Using both Cas13x and Cas13d variants, we achieved significant *MGMT* downregulation in established glioma cell lines and primary patient-derived gliomaspheres. While the concept of inhibiting *MGMT* to enhance TMZ efficacy is well-established, this study is among the first to apply the direct RNA targeting CRISPR-Cas13x and Cas13d variants for this purpose in clinically relevant primary patient-derived glioma models [4, 16, 25]. This novel RNA-guided RNA knockdown strategy induced TMZ sensitivity, demonstrating proof-of-principle for TMZ refractory *MGMT* unmethylated glioblastoma.

The Cas13-based RNA regulatory approach offers several advantages over other gene-targeting technologies. Unlike permanent genomic editing with Cas9, which carries the risk of irreversible off-target DNA mutations, Cas13 targets and degrades transient mRNA transcripts, making its effects reversible with a potentially more favorable safety profile [5, 15]. The Cas13 system is more direct and rapid compared to the multi-step dCas9-based epigenetic editing [11, 23, 26]. Furthermore, the smaller size of Cas13 variants like Cas13x, compared to the bulky dCas9-fusion proteins, may facilitate the development of optimized delivery systems that cross the blood-brain-barrier [20]. Additionally, so long as the designed guide RNAs have no sequence homology outside of the intended target genes, both Cas13x and Cas13d have been shown to exhibit little collateral activity, especially when compared to alternatives like RNAi and the use of small-molecule inhibitors [16].

Despite this proof-of-principle, our study has several limitations. The most significant challenge remains in vitro and in vivo delivery. Although lentivirus successfully established stable in vitro knockdown, such transduction strategies are unsuitable for clinical brain translation. Difficulties in delivering Cas13x RNPs to gliomasphere models highlight the need for safe, efficient, and tumor-restricted delivery vehicles (e.g., lipid nanoparticles or adeno-associated viruses) that can cross the blood-brain barrier [25, 27–29]. Notably, our study lacks in vivo validation in an orthotopic glioma xenograft model that would further establish translational efficacy, delivery, and safety of this system. In addition, while we did not identify any off target matches to our *MGMT* crRNAs without introducing features known to interfere with Cas13 activation (i.e., large mismatches or indels) [17] a comprehensive differential gene expression analysis with RNA-Seq will be necessary to confirm the absence of collateral activity in this system before its use in vivo and beyond.

Secondly, a notable observation in this study is that measurable chemosensitization was primarily achieved at TMZ concentrations (100–200 µM). While these levels exceed the typical steady-state concentrations found in patient CSF (~ 5–15 µM), they are consistent with established in vitro glioblastoma research standards. A systematic review by Poon et al. (2021) of over 212 studies found that IC50 values for glioma lines are frequently reported in the range of 200 µM to 1000 µM [30]. Furthermore, while the utilization of other Cas13 variants may enhance the potency of TMZ chemosensitization, we sought to characterize “on target only” variants to maximize the safety margin for future clinical application in the brain and to exploit the well-characterized *MGMT* methylation clinical biomarker in GBM.

Lastly, this study did not evaluate the effectiveness of the Cas13 system within the context of mismatch repair (MMR) deficiency. While *MGMT* promoter methylation remains the gold-standard biomarker for TMZ response, MMR expression is required to translate severe DNA damage into apoptosis [31]. Furthermore, at initial diagnosis, MMR deficiency is found in only ~ 4% of GBM cases and is often an acquired resistance mechanism occurring in ~ 25% of recurrent GBM [32]. Accordingly, our study only assessed primary cell lines from newly diagnosed *MGMT*-unmethylated glioma patients with confirmed MMR proficiency [21]. Future clinical implementation may benefit from the use of MMR proficiency as a secondary biomarker of therapeutic response.

In conclusion, CRISPR-Cas13-mediated *MGMT* RNA regulation is a novel strategy for MGMT-mediated chemoresistance. The speed, efficiency, and specificity of the Cas13 system make it a promising alternative to previous MGMT-targeting methods. While significant challenges, particularly in vivo delivery, remain, our findings provide a strong rationale for the continued development of Cas13-based therapeutics to overcome chemoresistance in glioblastoma.

## Supplementary Information

Below is the link to the electronic supplementary material.

Supplementary Material 1

Supplementary Material 2

## Data Availability

No datasets were generated or analysed during the current study.

## References

[1] Price M, Ballard C, Benedetti J, Neff C, Cioffi G, Waite KA, Kruchko C, Barnholtz-Sloan JS, Ostrom QT (2024, Oct 6) CBTRUS statistical report: primary brain and other central nervous system tumors diagnosed in the United States in 2017–2021. Neurooncology 26. 10.1093/neuonc/noae145 10.1093/neuonc/noae145 PMC11456825 39371035

[2] Stupp R, Mason WP, van den Bent MJ, Weller M, Fisher B, Taphoorn MJB, Belanger K, Brandes AA, Marosi C, Bogdahn U, Curschmann J, Janzer RC, Ludwin SK, Gorlia T, Allgeier A, Lacombe D, Cairncross JG, Eisenhauer E, Mirimanoff RO (2005 Mar 10) Radiotherapy plus concomitant and adjuvant temozolomide for glioblastoma. N Engl J Med 352. 10.1056/NEJMoa043330 10.1056/NEJMoa043330 15758009

[3] Stupp R, Hegi ME, Gilbert MR, Chakravarti A (2007 Sep 10) Chemoradiotherapy in malignant glioma: standard of care and future directions. J Clin Oncol 25. 10.1200/JCO.2007.11.8554 10.1200/JCO.2007.11.8554 17827463

[4] Gerson SL (2004 Apr) MGMT: its role in cancer aetiology and cancer therapeutics. Nat Reviews Cancer 4:4. 10.1038/nrc1319 10.1038/nrc1319 15057289

[5] Hegi ME, Diserens A-C, Gorlia T, Hamou M-F, de Tribolet N, Weller M, Kros JM, Hainfellner JA, Mason W, Mariani L, Bromberg JEC, Hau P, Mirimanoff RO, Cairncross JG, Janzer RC, Stupp R (2005 Mar 10) MGMT gene silencing and benefit from temozolomide in glioblastoma. N Engl J Med 352. 10.1056/NEJMoa043331 10.1056/NEJMoa043331 15758010

[6] Kato T, Natsume A, Toda H, Iwamizu H, Sugita T, Hachisu R, Watanabe R, Yuki K, Motomura K, Bankiewicz K, Wakabayashi T (2010 Nov) Efficient delivery of liposome-mediated MGMT-siRNA reinforces the cytotoxity of temozolomide in GBM-initiating cells - PubMed. Gene Ther 17. 10.1038/gt.2010.88 10.1038/gt.2010.88 20520650

[7] Zhang W, Zhang J, Hoadley K, Kushwaha D, Ramakrishnan V, Li S, Kang C, You Y, Jiang C, Song SW, Jiang T, Chen CC (2012 Jun 1) miR-181d: a predictive glioblastoma biomarker that downregulates MGMT expression. Neurooncology 14. 10.1093/neuonc/nos089 10.1093/neuonc/nos089 PMC3367855 22570426

[8] Nie E, Jin X, Wu W, Yu T, Zhou X, Shi Z, Zhang J, Liu N, You Y (2017 Apr 19) MiR-198 enhances temozolomide sensitivity in glioblastoma by targeting MGMT. J Neuro-Oncology 133:1. 10.1007/s11060-017-2425-9 10.1007/s11060-017-2425-9 28425046

[9] Quinn JA, Jiang SX, Reardon DA, Desjardins A, Vredenburgh JJ, Rich JN, Gururangan S, Friedman AH, Bigner DD, Sampson JH, McLendon RE, Herndon JE, Walker A, Friedman HS (2009 Oct 1) Phase I trial of temozolomide plus O6-benzylguanine 5-day regimen with recurrent malignant glioma. Neurooncology 11. 10.1215/15228517-2009-007 10.1215/15228517-2009-007 PMC2765345 19289491

[10] Gilbert MR, Wang M, Aldape KD, Stupp R, Hegi ME, Jaeckle KA, Armstrong TS, Wefel JS, Won M, Blumenthal DT, Mahajan A, Schultz CJ, Erridge S, Baumert B, Hopkins KI, Tzuk-Shina T, Brown PD, Chakravarti A, CurranJr WJ, Mehta MP (2013 Nov 10) Dose-dense temozolomide for newly diagnosed glioblastoma: a randomized phase III clinical trial. J Clin Oncol 31. 10.1200/JCO.2013.49.6968 10.1200/JCO.2013.49.6968 PMC3816958 24101040

[11] Zapanta Rinonos S, Li T, Pianka ST, Prins TJ, Eldred BSC, Kevan BM, Liau LM, Nghiemphu PL, Cloughesy TF, Lai A (2024 Jan 15) dCas9/CRISPR-based methylation of O-6-methylguanine-DNA methyltransferase enhances chemosensitivity to temozolomide in malignant glioma. J Neuro-Oncology 166:1. 10.1007/s11060-023-04531-z 10.1007/s11060-023-04531-z PMC10824881 38224404

[12] Han X, Abdallah MOE, Breuer P, Stahl F, Bakhit Y, Potthoff A-L, Pregler BEF, Schneider M, Waha A, Wüllner U, Evert BO (2023 Oct 1) Downregulation of MGMT expression by targeted editing of DNA methylation enhances temozolomide sensitivity in glioblastoma. Neoplasia 44. 10.1016/j.neo.2023.100929 10.1016/j.neo.2023.100929 PMC10475512 37634280

[13] Yousefi Y, Nejati R, Eslahi A, Alizadeh F, Farrokhi S, Asoodeh A, Mojarrad M (2024 May 19) Enhancing temozolomide (TMZ) chemosensitivity using CRISPR-dCas9-mediated downregulation of O6-methylguanine DNA methyltransferase (MGMT). J Neuro-Oncology 169:1. 10.1007/s11060-024-04708-0 10.1007/s11060-024-04708-0 38762829

[14] Lin K, Zou C, Hubbard A, Sengelmann S, Goudy L, Wang I-C, Sharma R, Pak J, Foster K, Ozawa T, de Groot JF, Phillips J, Vasudevan HN, Raleigh DR, Marson A, Murthy N, Gilbert LA, Berger MS, Liu SJ (2025 Jul 30) Multiplexed epigenetic memory editing using CRISPRoff sensitizes glioblastoma to chemotherapy. Neurooncology 27. 10.1093/neuonc/noaf055 10.1093/neuonc/noaf055 PMC12309708 39998382

[15] Villiger L, Joung J, Koblan L, Weissman J, Abudayyeh OO, Gootenberg JS (2024 Feb 2) CRISPR technologies for genome, epigenome and transcriptome editing. Nat Rev Mol Cell Biol 25:6. 10.1038/s41580-023-00697-6 10.1038/s41580-023-00697-6 38308006

[16] Abudayyeh OO, Gootenberg JS, Essletzbichler P, Han S, Joung J, Belanto JJ, Verdine V, Cox DBT, Kellner MJ, Regev A, Lander ES, Voytas DF, Ting AY, Zhang F (2017 Oct 4) RNA targeting with CRISPR–Cas13. Nat 550:7675. 10.1038/nature24049 10.1038/nature24049 PMC5706658 28976959

[17] Tong H, Huang J, Xiao Q, He B, Dong X, Liu Y, Yang X, Han D, Wang Z, Wang X, Ying W, Zhang R, Wei Y, Xu C, Zhou Y, Li Y, Cai M, Wang Q, Xue M, Li G, Fang K, Zhang H, Yang H (2022 Aug 11) High-fidelity Cas13 variants for targeted RNA degradation with minimal collateral effects. Nature Biotechnology 41:1. 10.1038/s41587-022-01419-7 10.1038/s41587-022-01419-7 35953673

[18] Reuter JS, Mathews DH (2010) RNAstructure: software for RNA secondary structure prediction and analysis. BMC Bioinformatics 11:129. 10.1186/1471-2105-11-129 20230624 10.1186/1471-2105-11-129 PMC2984261

[19] Wessels H-H, Méndez-Mancilla A, Guo X, Legut M, Daniloski Z, Sanjana NE (2020 Mar 16) Massively parallel Cas13 screens reveal principles for guide RNA design. Nat Biotechnol 38:6. 10.1038/s41587-020-0456-9 10.1038/s41587-020-0456-9 PMC7294996 32518401

[20] Konermann S, Lotfy P, Brideau NJ, Oki J, Shokhirev MN, Hsu PD (2018 Apr 19) Transcriptome engineering with RNA-targeting type VI-D CRISPR Effectors. Cell 173. 10.1016/j.cell.2018.02.033 10.1016/j.cell.2018.02.033 PMC5910255 29551272

[21] Minami JK, Morrow D, Bayley NA, Fernandez EG, Salinas JJ, Tse C, Zhu H, Su B, Plawat R, Jones A, Sammarco A, Liau LM, Graeber TG, Williams KJ, Cloughesy TF, Dixon SJ, Bensinger SJ, Nathanson DA (2023 Jun 12) CDKN2A deletion remodels lipid metabolism to prime glioblastoma for ferroptosis. Cancer Cell 41. 10.1016/j.ccell.2023.05.001 10.1016/j.ccell.2023.05.001 PMC10330677 37236196

[22] Pianka ST, Li T, Prins TJ, Eldred BSC, Kevan BM, Liang H, Zapanta Rinonos S, Kornblum HI, Nathanson DA, Pellegrini M, Liau LM, Nghiemphu PL, Cloughesy TF, Lai A (2024 Mar 1) D-2-HG Inhibits IDH1mut Glioma Growth via FTO Inhibition and Resultant m6A Hypermethylation. Cancer Res Commun 4. 10.1158/2767-9764.CRC-23-0271 10.1158/2767-9764.CRC-23-0271 PMC10959073 38445960

[23] Yang H, Patel DJ (2024 May 3) Structures, mechanisms and applications of RNA-centric CRISPR–Cas13. Nat Chem Biology 20:6. 10.1038/s41589-024-01593-6 10.1038/s41589-024-01593-6 PMC11375968 38702571

[24] Hussein M, Liu Y, Vink M, Kroon PZ, Das AT, Berkhout B, Herrera-Carrillo E (2024 Sep 10) Evaluation of the effect of RNA secondary structure on Cas13d-mediated target RNA cleavage. Mol Therapy - Nucleic Acids 35. 10.1016/j.omtn.2024.102278 10.1016/j.omtn.2024.102278 PMC11364014 39220269

[25] Rouatbi N, Walters AA, Costa PM, Qin Y, Liam-Or R, Grant V, Pollard SM, Wang JT-W, Al-Jamal KT (2024 Nov 1) RNA lipid nanoparticles as efficient in vivo CRISPR-Cas9 gene editing tool for therapeutic target validation in glioblastoma cancer stem cells. J Controlled Release 375. 10.1016/j.jconrel.2024.09.019 10.1016/j.jconrel.2024.09.019 39284526

[26] Pulecio J, Verma N, Mejía-Ramírez E, Huangfu D, Raya A (2017 Oct 5) CRISPR/Cas9-based engineering of the epigenome. Cell Stem Cell 21. 10.1016/j.stem.2017.09.006 10.1016/j.stem.2017.09.006 PMC6205890 28985525

[27] Rosenblum D, Gutkin A, Kedmi R, Ramishetti S, Veiga N, Jacobi AM, Schubert MS, Friedmann-Morvinski D, Cohen ZR, Behlke MA, Lieberman J, Peer D (2020 Nov) CRISPR-Cas9 genome editing using targeted lipid nanoparticles for cancer therapy. Sci Adv. 10.1126/sciadv.abc9450 10.1126/sciadv.abc9450 PMC7673804 33208369

[28] Wang C, Xue Y, Markovic T, Li H, Wang S, Zhong Y, Du S, Zhang Y, Hou X, Yu Y, Liu Z, Tian M, Kang DD, Wang L, Guo K, Cao D, Yan J, Deng B, McComb DW, Parsons RE, Minier-Toribio AM, Holt LM, Pan J, Hashemi A, Kopell BH, Charney AW, Nestler EJ, Peng PC, Dong Y (2025 Feb 17) Blood–brain-barrier-crossing lipid nanoparticles for mRNA delivery to the central nervous system. Nat Mater. 10.1038/s41563-024-02114-5 39962245 10.1038/s41563-024-02114-5 PMC12514551

[29] Chow RD, Guzman CD, Wang G, Schmidt F, Youngblood MW, Ye L, Errami Y, Dong MB, Martinez MA, Zhang S, Renauer P, Bilguvar K, Gunel M, Sharp PA, Zhang F, Platt RJ, Chen S (2017 Aug) AAV-mediated direct in vivo CRISPR screen identifies functional suppressors in glioblastoma. Nat Neurosci 14. 10.1038/nn.4620 10.1038/nn.4620 PMC5614841 28805815

[30] Poon MTC, Bruce M, Simpson JE, Hannan CJ, Brennan PM, Poon MTC, Bruce M, Simpson JE, Hannan CJ, Brennan PM (2021 Nov 18) Temozolomide sensitivity of malignant glioma cell lines – a systematic review assessing consistencies between in vitro studies. BMC Cancer 21:1. 10.1186/s12885-021-08972-5 10.1186/s12885-021-08972-5 PMC8600737 34794398

[31] Li GM (2008) Mechanisms and functions of DNA mismatch repair. Cell Res 18:85–98. 10.1038/cr.2007.115 18157157 10.1038/cr.2007.115

[32] Indraccolo S, Lombardi G, Fassan M, Pasqualini L, Giunco S, Marcato R, Gasparini A, Candiotto C, Nalio S, Fiduccia P, Fanelli GN, Pambuku A, Della Puppa A, D’Avella D, Bonaldi L, Gardiman MP, Bertorelle R, De Rossi A, Zagonel V (2019 Mar 15) Genetic, epigenetic, and immunologic profiling of MMR-deficient relapsed glioblastoma. Clin Cancer Res 25. 10.1158/1078-0432.CCR-18-1892 10.1158/1078-0432.CCR-18-1892 30514778

