# Supplementary material for: MGMT downregulation by CRISPR/Cas13 RNA-guided RNA targeting enhances glioma cell sensitivity to TMZ chemotherapy
Source: J Neurooncol. 2026 Mar 12;177(1):48. doi: 10.1007/s11060-026-05500-y (PMC12982228; doi:10.1007/s11060-026-05500-y)
Supplement: Supplementary file 1 — Supplementary Material 1 [file 11060_2026_5500_MOESM1_ESM.pdf]

Table S1. DNA templates of MGMT crRNAs for Cas13x All-in-one vector cloning

**crRNA-1**	Forward	5'-ACCGCGCAAACGGTGCGCACCGCGAGG-3'
	Reverse	5'-CAGCCCTCGCGGTGCGCACCGTTTGCG-3'
**crRNA-2**	Forward	5'-ACCGACAATCCTTGTCCATTTTTCCAA-3'
	Reverse	5'-CAGCTTGGAAAA**ATG**GACAAGGATTGT-3'
**crRNA-3**	Forward	5'-ACCGCTGGGACCTCCACGGCATCAGCT-3'
	Reverse	5'-CAGCAGCTGATGCCGTGGAGGTCCCAG-3'
**crRNA-4**	Forward	5'-ACCGGCCTGCCAGGGCTGCTAATTGCT-3'
	Reverse	5'-CAGCAGCAATTAGCAGCCCTGGCAGGC-3'
**NSC-crRNA**	Forward	5'-ACCGGTATTACTGATATTGGTGGG-3'
	Reverse	5'-CAGCCCCACCAATATCAGTAATAC-3

Note: Red letters are cloning sequence. Black letters are MGMT mRNA targeting sequence

Table S2. DNA templates of MGMT crRNAs for Cas13d All-in-one vector cloning

**crRNA-1**	Forward	5'-AAACCGCAAACGGTGCGCACCGCGAGG-3'
	Reverse	5'-AAAACCTCGCGGTGCGCACCGTTTGCG-3'
**crRNA-2**	Forward	5'-AAACACAATCCTTGTCCATTTTTCCAA-3'
	Reverse	5'-AAAATTGGAAAAATGGACAAGGATTGT-3'
**crRNA-3**	Forward	5'-AAACCTGGGACCTCCACGGCATCAGCT-3'
	Reverse	5'-AAAAAGCTGATGCCGTGGAGGTCCCAG-3'
**crRNA-4**	Forward	5'-AAACGCCTGCCAGGGCTGCTAATTGCT-3'
	Reverse	5'-AAAAAGCAATTAGCAGCCCTGGCAGGC-3'
**NSC-crRNA**	Forward	5'-AAACGTATTACTGATATTGGTGGG-3'
	Reverse	5'-AAAACCCACCAATATCAGTAATAC-3

Note: Red letters are cloning sequence. Black letters are MGMT mRNA targeting sequence

Table S3. Primers of crRNA DNA template synthesis for in vitro crRNA transcription for Cas13x

**crRNA-1**	Forward	5'-GCGGCCTCTAATAGGACTCACTATAGGGCGCAAACGGTGCGCACCGCGAGG-3
**crRNA-2**	Forward	5'-GCGGCCTCTAATAGGACTCACTATAGGGACAATCCTTGTCCATTTTTCCAA-3'
**crRNA-3**	Forward	5'-GCGGCCTCTAATAGGACTCACTATAGGGCTGGGACCTCCACGGCATCAGCT-3'
**crRNA-4**	Forward	5'-GCGGCCTCTAATAGGACTCACTATAGGGGCCTGCCAGGGCTGCTAATTGCT-3'
**NSC-crRNA**	Forward	5'-GCGGCCTCTAATAGGACTCACTATAGGGGTATTACTGATATTGGTGGG-3'
**drRNA scaffold**	Reverse	5'-AGCTGTAATCACCCCACAAA-3'

Note: Red letters are T7 promoter sequence. Black letters are MGMT mRNA targeting sequence. To synthesize each MGMT crRNA DNA templates, PCR reactions were employed with Cas13x-crRNA-All-in-One plasmids used as PCR template and primer pairs of each forward primer plus drRNA scaffold reverse primer.
