# Supplementary material for: MGMT downregulation by CRISPR/Cas13 RNA-guided RNA targeting enhances glioma cell sensitivity to TMZ chemotherapy
Source: J Neurooncol. 2026 Mar 12;177(1):48. doi: 10.1007/s11060-026-05500-y (PMC12982228; doi:10.1007/s11060-026-05500-y)
Supplement: Supplementary file 2 — Supplementary Material 2 [file 11060_2026_5500_MOESM2_ESM.pdf]

Reagent	Manufacturer	Catalog No.
Plasmid Reagents		
pCMV-Cas13X.1 plasmid	Addgene	171379
pLentiRNACRISPR_006	Addgene	138148
PCR Reagents		
BigDye Terminator v3.1 chemistry	Applied Biosystems	4336917
HiScribe T7 High Yield RNA Synthesis Kit	New England Biolabs	E2040S
Ni Spin Column	New England Biolabs	S1427S
REDTaq® ReadyMIX PCR Reaction Kit	Sigma-Aldrich	R2523-100RXN
SuperScript™ III Reverse Transcriptase	Invitrogen	56575
SYPRO™ Ruby Protein Blot Stain	Invitrogen	S4942-200ML
TRIzol™	Invitrogen	15596-018
Cell Culture & Experiments Reagents		
Antibiotic-Antimycotic (Anti-Anti, 100X)	GIBCO	15240-062
B27	GIBCO	12587010
DMEM	GIBCO	11995-065
EGF	Millipore	GF003
bFGF	Millipore	GF316
Heparin	Sigma-Aldrich	H3149
Lipofectamine™ CRISPRMAX™ Reagent	Invitrogen	CMAX00003
L-Glutamine	GIBCO	25030081
Mouse Anti-GAPDH	Proteintech	60004-I-Ig
Mouse Anti-MGMT	ThermoFisher	35-7000
MTT (3-(4,5-Dimethylthiazol-2-yl)-2,5-Diphenyltetrazolium Bromide)	Invitrogen	M6494
Neurobasal medium	GIBCO	21103-049
N2 Supplement	GIBCO	17502-048
Penicillin Streptomycin (Pen Strep)	GIBCO	15140-122
Rabbit Anti-HA	Sigma	H6908-100mL
Rabbit Anti-His	Proteintech	1000-0-AP
RNeasy® Mini Kit	Qiagen	74106
X-tremeGENE™ HP DNA Transfection Reagent	Roche	06366236001

Table S4. List of reagents used with manufacturer and catalog numbers.
